# A fast method for breeding by design via G × E interactions detected in large-scale climatic, phenomic and genomic data

**DOI:** 10.1093/nsr/nwag095

**Published:** 2026-02-11

**Authors:** Jing-Tian Wang, Xue-Lian Han, Miao-Miao Zhao, Han-Qing Zhang, Ying Chen, Qiu-Yun Jiang, Yuan-Ming Zhang

**Affiliations:** College of Plant Science and Technology, Huazhong Agricultural University, Wuhan 430070, China; College of Plant Science and Technology, Huazhong Agricultural University, Wuhan 430070, China; College of Plant Science and Technology, Huazhong Agricultural University, Wuhan 430070, China; College of Plant Science and Technology, Huazhong Agricultural University, Wuhan 430070, China; College of Plant Science and Technology, Huazhong Agricultural University, Wuhan 430070, China; College of Plant Science and Technology, Huazhong Agricultural University, Wuhan 430070, China; College of Plant Science and Technology, Huazhong Agricultural University, Wuhan 430070, China

**Keywords:** genome-wide association study, gene-by-meteorological factor interaction, machine learning, large-scale data, crops, climate change

## Abstract

Although large-scale populations are used to detect genes for polygenic traits, few studies integrate genes and gene-by-environment interactions (GEIs) into breeding by design. Here, we present Fast3VmrMLM, which uses eight big-data techniques to analyze climatic, phenomic and genomic data together to detect GEIs, decipher plasticity and guide breeding. In multi-environment joint analysis (MEJA) of maize, rice and soybean datasets, a total of 396 known genes and 84 known GEIs validated Fast3VmrMLM. In a 12-environment maize dataset, six GEIs interacting with five meteorological factors and two MEJA-detected GEIs helped to explain flowering time plasticity. Thirteen known genes, eight known GEIs and seven plasticity genes advanced flowering by 1.10–6.61 days, whereas nine known genes, one known GEI and three plasticity genes increased yield by 0.51–3.56 Mg·ha^−1^, identifying 15 high breeding potential hybrids and 29 genes. By incorporating single nucleotide polymorphisms, haplotypes and structural variations, Fast3VmrMLM offers a big-data platform for identifying GEIs and developing climate-adaptive cultivars.

## INTRODUCTION

Gene-by-environment interactions (GEIs) refer to the genes that contribute to phenotypes that differ among environments [1]. GEIs are recognized as a key determinant of organism phenotype and are crucial for genetic analysis [2,3], as well as being important for breeders seeking to improve agricultural production in specific regions [4]. Furthermore, dissecting the genetic basis is crucial for understanding the environmental adaptability of cultivars under climate change [5–7]. However, identifying GEIs remains challenging, particularly for polygenic traits [8,9]. Therefore, identifying GEIs and deciphering phenotypic plasticity are crucial for crop breeding and human genetics in the era of global climate change, highlighting the necessity of novel statistical methodologies [8].

Over the past two decades, genome-wide association studies (GWAS) have become a widely used method for identifying quantitative trait nucleotides (QTNs) and their associated genes for complex traits [10]. While these methods were not originally intended for identifying QTN-by-environment interactions (QEIs) and their GEIs [11,12], several indirect and direct methods have since been developed. Indirect methods use phenotypes across environments to generate indicators for conventional GWAS [2,13–15], but their statistical power is constrained by population size [10,16]. Direct methods incorporate all phenotypes across environments within a multi-trait-based [17] or QEI-based [18,19] mixed linear model (MLM). However, these models only consider the allelic substitution effect (*α*), the *α*-by-environment (*αe*) interaction effect, and their polygenic backgrounds. To address these limitations, Li *et al.* [20] established the 3VmrMLM method under a compressed variance component mixed model that considers all possible effects and controls for all potential polygenic backgrounds, thereby enabling more comprehensive detection of effects.

In the era of big data and artificial intelligence (AI), new algorithms are needed to handle large samples [10] and exploit AI technology applications [21]. Although several low-cost methods have been developed to identify QEIs [18,22] in large-scale human datasets [23], most of them [24–26] rely on an *α*-based model and therefore overlook dominant or small *α* loci [20,27]. Furthermore, their reliance on a sparse kinship matrix to reduce computational cost renders them unsuitable for crop association mapping populations. Therefore, algorithms that can handle large samples and numerous environments simultaneously, while enhancing QEI detection power, are essential.

To address the above issues, we incorporated eight statistical and computational technologies into the previous 3VmrMLM algorithm [20], creating a new, fast, efficient and large-scale algorithm named Fast3VmrMLM. This new algorithm can be used to directly identify QEIs in a large association mapping population across numerous environments. To dissect phenotypic plasticity, the regression intercepts and slopes of trait phenotypes on meteorological factors across multiple environments were used to indirectly identify QTN-by-meteorological factor interactions (QMIs). Furthermore, new modules were developed to detect the interactions between environments and bin/gene haplotypes (Fast3VmrMLM-Hap) or molecular markers, e.g. structural variants (SVs) and long non-coding RNA (lncRNA) types: Fast3VmrMLM-mQTL. The new methods were validated by simulations and applications in rice (a large population), maize (in numerous environments) and soybean (with SVs). In maize, eight GEIs explained flowering-time plasticity in response to temperature and solar radiation. The genes, GEIs and plasticity genes provide breeding by design (BBD) strategies under climate change. Our unified, low-cost software package provides a big-data platform for dissecting plasticity and improving environmental adaptability of cultivars.

## RESULTS

### Strategies for QEI detection and its utilization in deciphering the crop phenotypic plasticity

To overcome the limitations of existing methods for identifying QEIs, Fast3VmrMLM uses a full mixed model within a genome-wide scanning plus machine learning framework. Genome-wide scanning identifies the potential QEIs (pQEIs) by jointly modeling the effects of additive (*a*), dominant (*d*) and additive-by-environment (*ae*) and dominant-by-environment (*de*) interactions together with their polygenic backgrounds. To enable faster running, the 10 variance components in the full mixed model were compressed to three using the 3VmrMLM strategy [20]. The pQEIs are then incorporated into a multi-locus model, where their effects are estimated using a machine learning algorithm (EM empirical Bayes) [28]. Significant QTNs and QEIs are finally identified using likelihood ratio tests.

To address the challenges in runtime and memory under large population size (*n*), marker number (*m*) and environmental number (*n_e_*), eight statistical and computing technologies were integrated into the 3VmrMLM. Matrix inversion was performed iteratively using the preconditioned conjugate gradient (PCG) method [29]. In addition, rapid genome-wide scanning was enabled by a vectorized Wald test [30], together with parallel computation, conditional expectation [31] and the GRAMMAR-gamma approximation [32]. To improve memory efficiency, the *n* × *m* genotype matrix was stored in a binary data structure, kinship matrix calculation was performed by the PCG to eliminate large matrix, and Woodbury matrix transformation reduced the *nn_e_* × *nn_e_* variance–covariance matrix to a *q* × *q* matrix, where *q* is the number of non-zero effect markers, which is much smaller than *n_e_*. The Haseman–Elston (HE) regression [33] was used when the AI-REML algorithm [34] failed to converge.

To decipher the genetic basis of phenotypic plasticity, the regression intercepts and slopes of trait phenotypes on meteorological factors (e.g. photothermal time, PTT, °C·h) were used in the Fast3VmrMLM analysis [35]. Fast3VmrMLM-Hap extends single nucleotide polymorphisms (SNPs) to bin/gene haplotypes, while Fast3VmrMLM-mQTL incorporates SVs, lncRNA types and multi-allelic markers, enabling the indirect detection of QEIs using rare, structural and molecular variants.

### Identification of GEIs for yield-related traits in the 1439- and 18K-rice datasets

#### Identification of QEIs for 11 rice yield-related traits in multiple environments

To validate the Fast3VmrMLM and Fast3VmrMLM-Hap methods, 1439 hybrids (1 098 527 markers; two environments) [36] and 18K lines (2929 530 SNPs; three environments) [37] were re-analyzed to identify QTNs and QEIs for 11 rice yield-related traits (Table S1). These analyses used the top two principal components to control for their population structure.

Fast3VmrMLM identified 521 significant QTNs and 243 significant QEIs, as well as 75 suggested QEIs in the 1439 dataset. The 18K dataset yielded 310 significant QTNs, 218 significant QEIs and 18 suggested QEIs (Tables S2 and S3). Of these QEIs, 33.96% (108/318) and 1.27% (3/236) had a large dominant variance ratio (>25%), while 20.44% (65/318) and 31.78% (75/236), respectively, had small *αe* and *de* interaction effects (<0.10), indicating its advantages in identifying small *αe* and *de* QEIs.

Fast3VmrMLM-Hap identified 340 significant QTNs and 61 significant QEIs, as well as 23 suggested QEIs in the 1439 dataset. The 18K dataset yielded 479 significant QTNs, 264 significant QEIs and 8 suggested QEIs (Tables S3 and S4). Fast3VmrMLM-Hap identified a higher proportion of rare QEIs [minor allelic frequency (MAF) ≤ 3%] in the 18K dataset (161/272; 59.19%) than Fast3VmrMLM (12/236; 5.08%), indicating its advantage in identifying rare QEIs.

#### Identification of known GEIs for rice yield-related traits

**Figure 1.**
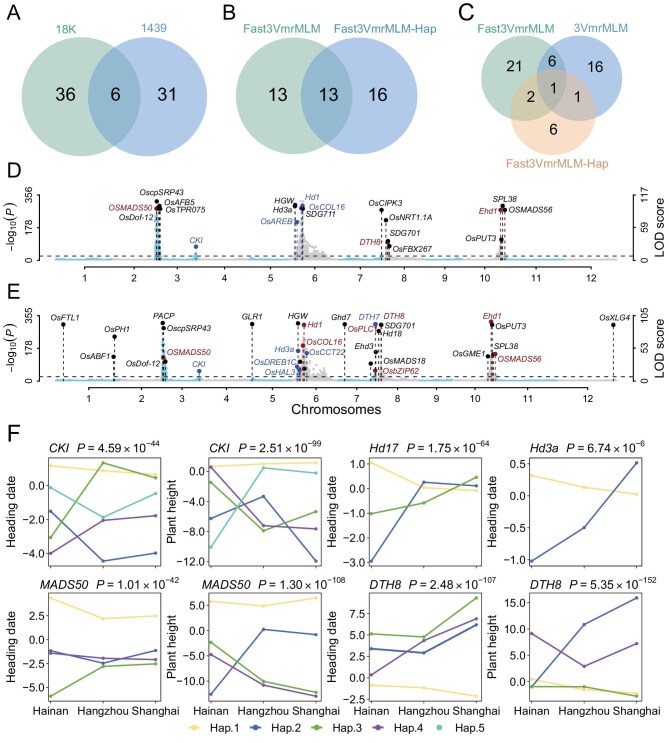
Known GEIs and Manhattan plots for heading date in the 18K-rice dataset [35]. (A) The number of GEIs around significant/suggested QEIs identified by the new methods in the 18K-rice and 1439-rice datasets. (B) The number of GEIs around significant/suggested QEIs identified by Fast3VmrMLM and Fast3VmrMLM-Hap in the 18K-rice dataset. (C) The number of GEIs around significant/suggested QEIs identified by Fast3VmrMLM, Fast3VmrMLM-Hap and 3VmrMLM in the 1439-rice dataset. (D and E) The *x*-axis represents the chromosomal and physical coordinates of markers, while the left *y*-axis represents the negative logarithmic transformation of the *P* value obtained from genome-wide scanning, and the right *y*-axis represents the LOD score obtained from the likelihood ratio test using the new methods (see Materials and methods section). Known genes around ±200 kb windows of significant QTNs and GEIs around significant QEIs (*P* value < 1.71e−8, shown as the dashed line) are marked in black (QTNs), blue (QEIs) and dark red (both QTNs and QEIs). The variants with LOD scores greater than 90 were transformed by ${\mathrm{LOD^{\prime} = }}\frac{{( {{\mathrm{LOD - 90}}} )}}{{100}} + 90$. (F) The gene haplotype (Hap) effects (*y*-axis) in three environments (*x*-axis) were calculated from the accession phenotypes corrected by polygenic background. The *P* values of haplotype-by-environment interaction in two-way ANOVA are shown in the top right.

Known genes with experimentally validated associations with yield-related traits at http://www.ricedata.cn/ were found within ±200 kb of the aforementioned QEIs (Table S5). Of these, genes previously reported to interact with meteorological factors or environmental conditions were defined as known GEIs [20], while genes without evidence of environmental interactions were defined as plasticity genes. Consequently, Fast3VmrMLM identified 30 and 26 known GEIs in the 1439- and 18K-rice datasets, respectively, while Fast3VmrMLM-Hap identified 10 and 29 known GEIs (Table S6, Fig. 1A–E). Two datasets commonly identified six known GEIs, and two methods commonly identified 16 known GEIs in the two datasets, indicating their repeatability (Table S6, Fig. 1A–C). For example, *DTH8* was identified by both new methods in both datasets and has been reported to respond to photoperiod [38]; Figure 1F shows the differences in haplotype effects across environments. The two new methods identified independently 35 and 22 known GEIs, indicating their complementarity.

#### Comparison of the new methods with existing methods

To demonstrate the superiority of the new methods, three existing methods were employed to re-analyze the 1439-rice dataset. In detail, 3VmrMLM re-analyzed the phenotypes in all environments to directly identify QEIs [20], whereas FarmCPU [12] and EMMAX [11] re-analyzed the trait differences between two environments to indirectly identify QEIs. Known GEIs were identified within ±200 kb windows of significant (*P* ≤ 4.55e–8) QEIs. The results showed that Fast3VmrMLM, Fast3VmrMLM-Hap, 3VmrMLM, FarmCPU and EMMAX identified 30, 8, 18, 5 and 3 GEIs, respectively (Table S6), indicating the superiority of 3VmrMLM-based methods compared to *α*-based methods. Six known GEIs were identified by both Fast3VmrMLM and 3VmrMLM, and one by Fast3VmrMLM-Hap and 3VmrMLM. Three known GEIs (*Hd1, DTH8* and *OsCCA1*) were identified by two new methods and two *α*-based methods (Table S6). All the genes identified by FarmCPU and EMMAX were also identified by the two new methods. The results suggest that the methods are repeatable and complementary.

### Deciphering the phenotypic plasticity of maize flowering time (FT) in a Genomes-to-Fields (G2F) dataset

#### The phenotypic plasticity analysis of maize FT

**Figure 2.**
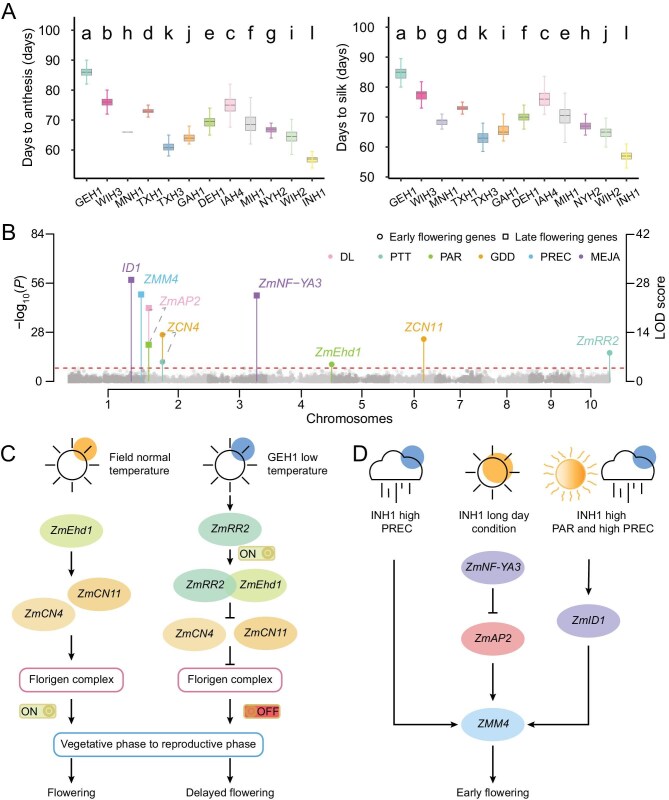
Deciphering the phenotypic plasticity of maize FT in a G2F dataset. (A) ANOVA and least significant difference (LSD) multiple comparisons (*α* = 0.05) for FT (*y*-axis) in 12 environments (*x*-axis). (B) Known GEIs for early (circular dot) and late (square) FT identified using both MEJA (purple) and the regression parameters of FT on meteorological factors, including DL, PTT, PAR, GDD and PREC. (C) Hypothetical molecular mechanism of delayed flowering regulation in GEH1. (D) Hypothetical molecular mechanism of early flowering regulation in INH1.

This study used the maize G2F dataset [39], which contains 1140 maize hybrids, each with 437 214 SNPs and FT phenotypes across 12 environments (Table S7). Analysis of variance (ANOVA) and multiple comparisons revealed a significant difference in FT across environments (*P* = 9.88e–324; Fig. 2A). Notably, the average days to anthesis (DTA) increased from 56.85 days in West Lafayette (INH1) to 86.45 days in Göttingen (GEH1), while the average days to silking (DTS) increased from 57.16 to 85.05 days (Fig. 2A), demonstrating the phenotypic plasticity of maize FT.

Five meteorological factors were considered in these environments (Text S1). GEH1 had the lowest accumulated temperature (2.90 °C·day) compared to the others (8.53). The photoperiod (12.89 h), photosynthetically active radiation (PAR; 121.18 MJ m^−2^ day^−1^) and precipitation (PREC; 9.08 mm) in INH1 differed from those in the other environments (12.07, 111.06 and 3.72), indicating differences in the meteorological factors between the environments.

As for previous methods [2,40,41], the critical windows of each meteorological factor for FT were determined based on biological meaning [42] and correlation analysis (Text S1). The results are shown in Fig. S1.

#### Identifying QEIs, QMIs and their known GEIs for FT

To identify the FT QEIs/QMIs, we calculated the regression intercepts and slopes of trait phenotypes on meteorological factors within the selected windows (Table S8). We then associated these parameters with genetic markers to indirectly identify QMIs using Fast3VmrMLM and Fast3VmrMLM-Hap [9]. These two methods identified 235 and 249 significant QMIs, as well as 40 and 104 suggested QMIs, respectively (Table S9). All the phenotypes in 12 environments were also jointly analyzed by Fast3VmrMLM and Fast3VmrMLM-Hap, which directly identified 119 and 143 significant QTNs, respectively, as well as 75 and 108 significant QEIs (Table S9).

Eight previously reported FT regulators were discovered within 200 kb upstream and downstream of the aforementioned QEIs and QMIs (Table S10, Fig. 2B). In detail, Fast3VmrMLM indirectly identified *ZmRR2, ZCN4* and *ZmAP2*, which interact with the meteorological factors PTT, day length (DL) and PAR, respectively, and directly identified *ZmNF-YA3*. Meanwhile, Fast3VmrMLM-Hap indirectly identified *ZmEhd1, ZCN4, ZCN11* and *ZMM4*, which interact with PAR, growing degree days (GDD), GDD and precipitation (PREC), respectively, while directly identifying *ID1*.

All eight of the above GEIs have been reported to regulate FT [43–46], five of which (*ZmAP2, ID1, ZmRR2, ZmNF-YA3* and *ZMM4*) have been found to be regulated by meteorological factors [47–50] (Table S10). These GEIs could help to elucidate the reasons for late flowering in GEH1 and early flowering in INH1 (Fig. 2C–D; see the Discussion section).

### Identification of QEIs for soybean seed size between SVs and environments

To validate Fast3VmrMLM-mQTL in identifying molecular quantitative trait loci (QTLs) in a pan-genome dataset, it was used to associate 98 636 SVs with a seed size of 206 soybean accessions across 10 environments between 2008 and 2015 [51]. *Glyma.20G085100* was found within a ±150 kb window of a significant SV-by-environment interaction. This gene has previously been shown to be genuinely associated with soybean seed weight [52], and its CCT domain is known to play a key role in photoperiod-related adaptation in *Arabidopsis* [53].

### Monte Carlo simulation studies

#### Advantages of Fast3VmrMLM over other methods

**Figure 3.**
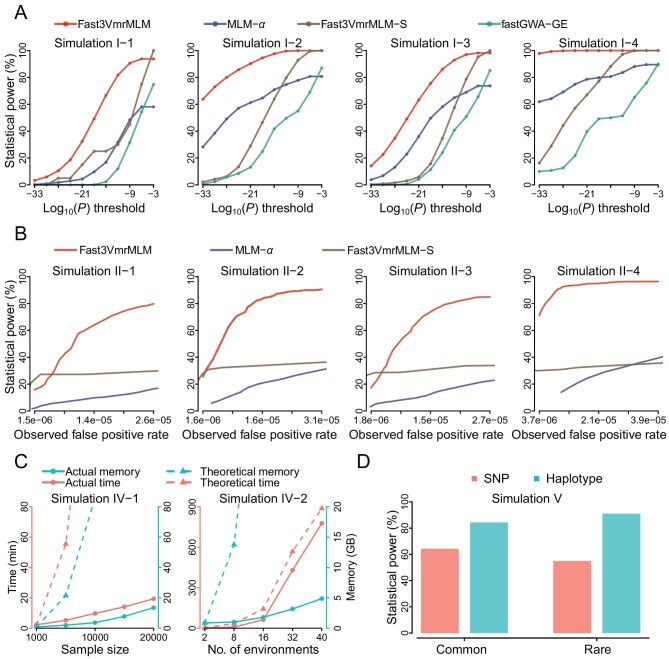
Comparison of different models/methods in Monte Carlo simulation studies. (A) Comparison of the average power (*y*-axis) of four mixed linear models at different *P* value thresholds (*x*-axis) in simulation datasets I-1 to I-4 (100 replicates). (B) Comparison of the average power (*y*-axis) of the Fast3VmrMLM with MLM-*α*, genome scanning alone (Fast3VmrMLM-S) at different observed false positive rates (*x*-axis) (100 replicates). (C) Running rimes and memory consumption (*y*-axis) of Fast3VmrMLM in different population sizes (left) and environmental numbers (right; *x*-axis; 3 replicates). (D) Comparison of the average power (*y*-axis) of Fast3VmrMLM and Fast3VmrMLM-Hap in identifying common and rare variants (100 replicates).

To confirm the advantages of the new Fast3VmrMLM method, simulation datasets I-1 to I-4 were analyzed. These datasets included 1000 individuals, each with 100 000 SNPs. The analysis was performed using four methods: Fast3VmrMLM, *α*-based MLM (MLM-*α*), genome scanning alone (Fast3VmrMLM-S) and fastGWA-GE [22]. These datasets were simulated using PLINK v1.9 [54], in which the phenotypes of two environments were obtained based on three QTNs, seven QEIs (*r*^2^: 5%–10%) and four types of polygenic background (*r*^2^: 0%–15%) (Table S11, Text S2). Under the Bonferroni-correction *P* value threshold of 5.0e−7, the average powers were 97.97%, 75.50%, 96.00% and 64.32% for the four aforementioned methods, respectively (Table S12). Their false positive rates (FPRs) were 0.05‱, 0.04‱, 0.50‱ and 0.09‱, respectively. Fast3VmrMLM achieved the highest average F_1_ score (0.97) in all four simulation datasets (I-1 to I-4) compared to the other methods (0.74–0.88) (Table S13). It also had the minimum mean absolute deviation (MAD) of effect estimates (0.46) compared to the other methods (5.22–8.48) (Table S14). All four methods had zero MAD for position estimates. Figure 3A shows the powers under different significance thresholds (1.0e−33 to 1.0e−3), demonstrating the superior performance of Fast3VmrMLM.

To further evaluate the performance of Fast3VmrMLM, a real genotypic dataset comprising 600 individuals, each with 100 000 SNPs, was randomly selected from the 1439-rice dataset [36] for simulation dataset II. The phenotypes in three environments were simulated in a manner similar to simulation in dataset I (Table S15, Text S2). Consequently, Fast3VmrMLM achieved the highest power (87.75%), the lowest FPR (0.3‱) and the highest F_1_ score (0.82) compared to the other methods (power: 2.40%–72.75%; FPR: 0.16–54.68‱; F_1_ score: 0.04–0.44; Tables S16 and S17). Fast3VmrMLM had a lower MAD for the effect estimates (1.62) than the other methods (5.69–13.65) (Table S18), and a relatively lower MAD for the position estimates (15.71 kb) than the other methods (Table S19). Figure 3B shows the powers and FPRs under different significance thresholds (1.0e−58 to 1.0e−1), demonstrating the same trend in simulation dataset I.

To evaluate the type I error rate of Fast3VmrMLM [27,35,55], a simulation dataset III of 600 individuals, each with 100 000 SNPs, was analyzed (Text S2). The phenotypes of the two environments were obtained based on polygenic backgrounds (*r*^2^: 10%) and normal error. Association tests were conducted for 10e+8 times in 1000 replicates. The observed type I error rate under the Bonferroni-correction threshold (*P* value < 5.0e−7) was 4.7e−7, which is close to the theoretical value of 5.0e−7, indicating no inflation or deflation of Fast3VmrMLM.

#### Effect of population sizes and the number of environments on running time and memory usage when using Fast3VmrMLM

First, to investigate the effect of population size and number of environments on the running time and memory usage of Fast3VmrMLM, a simulation dataset (IV-1) was obtained using PLINK v1.9 (Table S20, Text S2) with 1000, 5000, 10 000, 15 000 and 20 000 individuals, each with one million SNPs, in two environments. The corresponding running times were 2.22, 5.20, 9.74, 14.06 and 19.33 min, respectively, while the corresponding memory usage was 0.86, 1.94, 3.63, 7.73 and 13.51 GB (Fig. 3C). Second, simulation dataset IV-2 was obtained using PLINK v1.9 (Text S2, Table S20) with 1000 individuals, each with a million SNPs, in 2, 8, 16, 32 and 40 environments. The corresponding running times and memory usage were 2.22 min and 0.86 GB, 8.94 min and 1.02 GB, 62.14 min and 1.78 GB, 430.79 min and 3.17 GB, and 777.48 min and 4.88 GB, respectively (Fig. 3C). The same trends observed in previous studies were evident here.

In addition, a further simulation dataset (IV-3) was created to obtain the running time and memory usage of Fast3VmrMLM on a 60-CPU, 1 TB memory server. The first simulation took 48.01 h and used 22.70 GB of memory to analyze 20 000 individuals, each with one million SNPs, across 20 environments, while the second simulation took 82.83 h and used 50.27 GB of memory to analyze 5000 individuals, each with one million SNPs, across 40 environments (Table S20). In summary, Fast3VmrMLM can meet most requirements for joint analysis across multiple environments.

#### The advantage of Fast3VmrMLM-Hap in identifying rare variants

To evaluate the effectiveness of Fast3VmrMLM-Hap in identifying rare variants, a simulation dataset (V) involving 1136 Simmental beef cattle, each with 669 742 SNPs [56], was created. Their phenotypes in two environments were simulated using three QTNs and seven QEIs; two QTNs and two QEIs had MAFs of 3% and 1%, respectively (Table S21, Text S2). Fast3VmrMLM-Hap demonstrated greater power in identifying these MAF variants than Fast3VmrMLM (91.00% vs. 55.00%, respectively; Fig. 3D), indicating its superiority in identifying rare variants.

#### Availability of Fast3VmrMLM-mQTL in identifying molecular markers

A simulation dataset (VI) comprising 1000 individuals, each with 33 341 SVs, was created to evaluate Fast3VmrMLM-mQTL’s ability to identify molecular markers with any number of genotypes. The phenotypes in two environments were simulated using six causal SV-by-environment interactions and four causal SVs (*r*^2^: 5%–10%) (Table S22, Text S2). The average power was 98.00% and the FPR was 0.03‱ (Table S22), indicating its effectiveness in detecting SV variants.

## DISCUSSION

This study has made several significant advancements to QEI/GEI detection methodology and its applications. First, eight big-data statistical and computational techniques were incorporated into 3VmrMLM to create Fast3VmrMLM. This reduced the runtime by almost 50-fold in the 1439-rice dataset and enabled a GWAS with big data (18K-rice and maize G2F datasets), which is beyond the capacity of existing methods. This algorithm enables the rapid and cost-effective identification of QEIs in large populations and many environments. Using a 60-thread and 1-TB memory server, Fast3VmrMLM took: 19.33 min and 13.51 GB to analyze 20 000 cultivars, each with one million markers, in two environments; 777.48 min and 4.88 GB to analyze 1000 cultivars, each with one million markers, in 40 environments; and 48.01 h and 22.70 GB to analyze 20 000 cultivars, each with one million markers, in 20 environments. Fast3VmrMLM also outperforms a sparse matrix-based fastGWA-GE in speed (1.93 vs. 7.12 min) and memory (0.57 vs. 3.34 GB) in a real crop genomic dataset (simulation dataset II). This reduces the hardware requirements and enables the cheap running for multiple-environment joint analysis.

The regression parameters of trait phenotypes on environmental indicators are then used as phenotypes to indirectly identify QEIs using the QTN module of Fast3VmrMLM [35]. These environmental indicators include meteorological factors, treatment levels, fertilization levels and soil nutrient content. In this study, six known GEIs around QMIs help to explain maize FT plasticity.

Finally, Fast3VmrMLM effectively overcomes the limitations of existing methods when identifying dominant, small *α* and rare variants, thereby improving the QEI detection power. In simulation dataset II, Fast3VmrMLM achieved a higher power (87.75%) and a lower FPR (0.3‱) than other methods (2.40%–72.75% and 0.16‱–54.68‱, respectively; Tables S16 and S17). In the analysis of real data, the new methods identified 42, 37 and 10 known GEIs supported by biological experiments in the 1439-rice, 18K-rice and G2F-maize datasets, respectively (Tables S6 and S10, Figs 1 and 2B). This validates the effectiveness of the new methods. Fast3VmrMLM-mQTL was also developed to identify environmental interactions with molecular loci, including SVs, regulatory elements, lncRNA types, enhancers and multiple alleles. Its effectiveness was demonstrated in a simulation study (Table S22) and a soybean SV data analysis.

### Comparison of the new and existing methods

**Figure 4.**
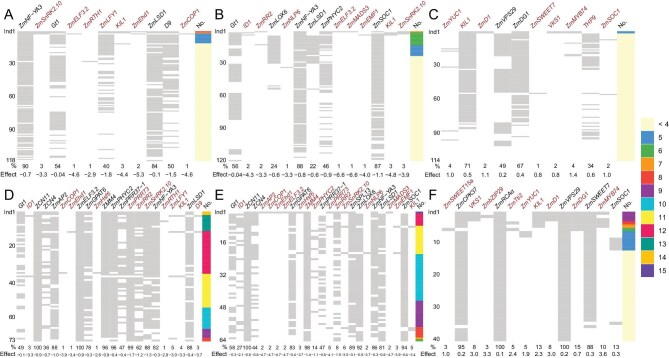
Distribution of superior haplotypes for known genes and GEIs in the top 10% of early-flowering and high-yield maize cultivars. (A–F) Each column indicates a known gene (A–C) or a known/plasticity gene (D–F) identified by new methods, while each row indicates a cultivar in the top 10% of early DTA (A and D), DTS (B and E) cultivars and GY (C and F). The genes with breeding potential are indicated by dark red. %, the proportion of each superior haplotype indicated by gray color and appearing in the top 10% of early-flowering or high-yield cultivars; Effect, the effect of superior haplotype; No., the number of the superior haplotypes of known/plasticity genes in each cultivar.

There are three key differences between the Fast3VmrMLM of Wang *et al.* [35] and the new method proposed in this study. First, the former performs single-environment analysis to identify QTNs, whereas the latter conducts a multi-environment joint analysis (MEJA) to simultaneously identify QTNs and QEIs. The former then uses the AI-REML algorithm to estimate the parameters in MLM iteratively. However, when the iteration does not converge, the FPR is high. To address this issue, HE regression was used in this study to estimate the parameters when AI-REML failed to converge. Finally, this study presented QMIs. These results are beneficial for explaining phenotypic plasticity (Fig. 2) and for identifying plasticity genes and germplasms (Fig. 4).

Both the 3VmrMLM [20] and the Fast3VmrMLM proposed in this study are used for MEJA to identify QTNs and QEIs simultaneously. However, there are three differences between them. First, unlike 3VmrMLM, Fast3VmrMLM uses eight big-data techniques to address runtime and memory challenges in GWAS of large association mapping populations across numerous environments. Therefore, Fast3VmrMLM is recommended for populations exceeding 4000 individuals or with more than five environments. Otherwise, the two methods can be used together (Fig. 1C, Table S6). Also, Fast3VmrMLM identifies QMIs. Finally, while 3VmrMLM uses SNPs, Fast3VmrMLM uses different marker types, including SNPs, bin/gene haplotypes and SVs.

### The potential of new methods for BBD of climate-adaptive cultivars

Peleman and van der Voort [57] introduced the concept of BBD to utilize all allelic variation in key agronomic genes. However, gene identification remains a major obstacle. To overcome this, extensive genomic selection research has been pursued [58–60]. While Wei *et al.* [37] used a large 18K-rice population to identify more genes, we present a new algorithm to identify more genes, GEIs and plasticity genes (Table S23, Fig. S2) for BBD.

To implement BBD, breeders have sought to construct a two-way table of cultivars and allelic effects. Here, we first identified 11, 14 and 10 known genes for DTA, DTS and grain yield (GY), respectively, using Fast3VmrMLM. Best linear unbiased prediction (BLUP) values across 12 environments were then used to estimate haplotype effects of these genes. Superior haplotypes associated with early flowering and high yield were identified, and their enrichment in the top 10% of high-performance cultivars for each trait (based on the BLUP values of DTA, DTS and GY) is shown in Fig. 4A–C. Seven DTA and eight DTS genes could advance flowering by 1.75–6.61 days, with utilization frequencies of 0.83%–39.83%. Meanwhile, eight GY genes could increase yield by 0.51–1.44 Mg·ha^–1^, with utilization frequencies of 0.88%–71.05%. Notably, 12 excellent hybrids were among the top 10% for both early flowering and high yield. For example, W10004_0241/PHP02 and W10004_0395/PHP02 exhibited high proportions of early-flowering and high-yield haplotypes at 45.45% and 36.36%, and 40.00% and 30.00%, respectively.

To further implement BBD under climate change, we incorporated 8 DTA, 8 DTS and 1 GY known GEIs, 9 DTA, 12 DTS and 5 GY plasticity genes, and 5 DTA, 4 DTS and 8 GY known genes into the BBD (Tables S10 and S23, Fig. 4). Phenotypic data from INH1 were used to estimate gene haplotype effects, leading to the identification of early-flowering and high-yield haplotypes in INH1. The top 10% of early-flowering and high-yield cultivars in INH1 are shown in Fig. 4D–F. Eleven DTA genes (4GEIs, 2 plasticity genes and 5 known genes) and 15 DTS genes (6 GEIs, 6 plasticity genes and 3 known genes) were found to advance flowering by 1.10–4.68 days. Meanwhile, nine GY genes (one GEIs, three plasticity genes, and five known genes) could increase yield by 0.69–3.56 Mg·ha^–1^. Notably, three hybrids (W10004_0032/PHK76, W10004_0956/PHK76 and W10004_0991/PHK76) were among the top 10% for both early flowering and high yield, exhibiting high proportions of early-flowering (45.45%–59.01%) and high-yield (28.57%) haplotypes.

These results demonstrate the potential of Fast3VmrMLM to provide environmentally stable superior haplotypes and cultivars under climate change. Given its high statistical power and compatibility with large-scale data (Fig. 3, Tables S6, S10 and S20), we expect this tool to facilitate further discoveries in the future.

### Known GEIs identified directly and indirectly in the G2F-maize dataset decipher maize FT plasticity

Unlike the identification of QMIs in previous studies [2,16,40,41], Fast3VmrMLM is capable of handling a large-scale G2F dataset. The results can be used to elucidate maize FT plasticity, as outlined below.

Delayed flowering in GEH1 may be explained by the interactions of *ZmRR2, ZmEhd1, ZmCN4* and *ZCN11* with PTT, PAR, GDD and PTT, respectively, as identified by new methods (Fig. 2B, Table S10). We found that the effective accumulated temperature during the early stage in GEH1 (2.90) was notably lower than in other regions (8.53). Hu *et al.* [50] demonstrated the upregulation of *ZmRR2* at low temperatures. Cho *et al.* [43] showed that *ZmRR1* interacts with *ZmEhd1* to repress its activity, thereby reducing the expression of downstream florigen genes such as *ZCN8*. Meanwhile, Deji *et al.* [61] observed a comparable expression pattern for *ZmRR2* and *ZmRR1*. Together, these findings suggest that *ZmRR2* may interact with *ZmEhd1* to form an inactive complex that suppresses the transcription of florigen-like genes (e.g. *ZCN8*). *ZCN4* and *ZCN11* are also florigen-like genes [46]. This would delay vegetative-to-reproductive transition in GEH1 during the early stages (Fig. 2C). However, direct experimental evidence of the interactions between *ZmEhd1, ZmCN4* and *ZCN11* at low temperatures is needed in future studies.

During the floral transition stage, interactions of *ZmAP2* with DL and PAR, and of *ZMM4* with PTT were detected using the new methods, while MEJA identified two GEIs (*ZmNF-YA3* and *ZmID1*) (Fig. 2B, Table S10). Therefore, early flowering in INH1 can be explained from three aspects. First, the photoperiod during the floral transition stage in INH1 (12.89 h) was longer than in other regions (12.07 h). Su *et al.* [49] demonstrated that long-day conditions induce the expression of *ZmNF-YA3*, which negatively regulates *ZmAP2* and subsequently promotes the expression of the floral organ gene *ZMM4* [45]. This would promote the vegetative-to-reproductive transition in INH1 under long-day conditions during the floral transition stage (Fig. 2D). We then found that PAR during the floral transition was higher (121.18) in INH1 than in other regions (111.06). Feng *et al.* [47] demonstrated that ultraviolet light upregulates *ZmID1*, while Danilevskaya *et al.* [45] confirmed that the overexpression of *ZMM4* in *id1* mutants suppresses their late-flowering phenotypes. This indicates that *ZMM4* likely functions downstream of *id1*. Together, these findings suggest that the higher PAR levels in INH1 during the floral transition may enhance the expression of *ZmID1*, thereby promoting the downstream expression of *ZMM4* and accelerating flowering (Fig. 2D). Finally, we found that PREC during the floral transition was higher in INH1 (9.08 mm) than in other regions (3.72 mm). Liu *et al.* [48] confirmed that the expression of the floral induction gene *ZMM4* was upregulated, indicating that higher PREC levels in INH1 during floral transition enhance *ZMM4* expression and promote flowering. In summary, the longer photoperiod, higher PAR and greater PREC levels in INH1 during floral transition collectively promote *ZMM4* activation, resulting in early flowering of maize in INH1 (Fig. 2D).

We found that TXH1 and GAH1 are located at lower latitudes than INH1. However, TXH1 and GAH1 had a larger DTA than INH1. This is due to lower GDD accumulation during the critical developmental period (Fig. S3), caused by the earlier planting in TXH1 (10 March) and GAH1 (25 March). This delayed the flowering in TXH1 and GAH1. Conversely, the later planting of INH1 (24 May) increased GDD accumulation, resulting in earlier flowering. This mechanism is similar to that observed for late flowering in GEH1 (Fig. 2C).

### Extension of SNP-based GWAS to haplotype-based GWAS

Current GWAS methods primarily identify SNP–trait associations in complex traits but exhibit limited power for detecting rare QTNs/QEIs [20,27,35,62] and are imprecise in pinpointing causal genes within large locus-derived intervals [63]. The availability of pan-genome data [64,65]—containing SVs, lncRNA types, regulatory elements, enhancers and multiple alleles—has proved to be valuable for genetic analysis [66,67], motivating gene- or haplotype-based GWAS methods [27,35,62]. Qi *et al.* [63] validated *GhFAH1*’s role in cotton fiber quality. Here, Fast3VmrMLM-Hap identified 7 (1439 dataset) and 16 (18K dataset) more known GEIs than Fast3VmrMLM (Table S6, Fig. 1B–C), including 2 and 13 rare loci (MAF ≤ 0.05). Simulations confirmed these benefits (Table S21). When applied to gene-based haplotypes for heading date in the 18K-rice dataset, Fast3VmrMLM-mQTL identified only three significant GEIs (*RFT1, OsNPF3.1, SDG701*), with evidence supporting only *RFT1* (Table S6), indicating a need for future refinement.

When applying the new method, three parameters may be adjusted, namely svpal, c_threshold and numofHaplotypes. First, the svpal was used to set the *P* value threshold for potential QTNs/QEIs. The recommended threshold depends on the population size; the larger the population size, the smaller the threshold. For example, svpal = 0.05/0.01 for a population size of less than 4000, and svpal = 1.0e−5 for a population size of more than 20 000. Second, the c_threshold and numOfHaplotypes parameters can be adjusted when constructing haplotypes in Fast3VmrMLM-Hap. The c_threshold (0 to 1) is used to determine bin markers; the smaller the value, the larger the number of bin markers [68]. The default value is 0.7. The numofHaplotypes parameter determines the maximum number of haplotypes. The default value is three.

Identifying epistasis is challenging due to the large number of marker pairs and the need for false positive control. Although some methods have been developed, only the epistasis between the QTNs with known/candidate genes [37] and marginal epistasis [69] have been detected. Therefore, identifying QTN-by-QTN interactions requires further research.

## MATERIALS AND METHODS

### Compressed variance component mixed model

Due to the substantial computational burden posed by 10 variance components in the full mixed model including environmental effects, additive (*a*) and dominant (*d*) effects, *ae* and *de* interaction effects, and their polygenic backgrounds (Text S3), we compressed them into three, as outlined by Li *et al.* [20].


(1) \begin{eqnarray*}
{{\bf y}} = {{\bf X{\boldsymbol \beta} }} + {{{\bf Z}}}_{i{\mathrm{GE}}}{{{\bf \gamma }}}_{i{\mathrm{GE}}} + {{{\bf U}}}_{{\mathrm{GE}}} + {{\boldsymbol \varepsilon }},
\end{eqnarray*}

where **X** is the design matrix for fixed effect **β**, including environmental effects; **Z**_*i*GE_ is the design matrix for the random vector **γ**_iGE_ of combination effects between the *i*th marker and environments; **U** ∼ MVN(0,*τ*_ge_**K**_GE_) is **γ**_*i*GE_-based polygenic background vectors, where *τ*_ge_ is polygenic variance and **K**_GE_ is kinship matric derived from **Z**_*i*GE_, as described by Li *et al.* [20]; residual error **ε** ∼ *N*(0,*τ***I**_n_e_n_), where *τ* is residual variance.

### Restricted maximum log-likelihood (REML) function and its solution

The REML function of Equation (1) was:


(2) \begin{eqnarray*}
{l}_R\!\left( {{\tau }_{{\mathrm{ge}}},\tau } \right) \propto &-& \frac{1}{2}\Big[ \left( {n - q} \right){\mathrm{log}}\left( {{\tau }_{{\mathrm{ge}}}} \right) + {\mathrm{log}}\left( {{\bf \Sigma }} \right)\\
&&+\, {\mathrm{log}}\left| {{{{\bf X}}}^{\mathrm{T}}{{{\bf \Sigma }}}^{ - 1}{{\bf X}}} \right| + \frac{1}{\tau }{{{\bf y}}}^{\mathrm{T}}{{\bf Py}} \Big], \\
\end{eqnarray*}

where **P** = **Σ**^−1^ − **Σ**^−1^**X(X***^T^*  **Σ**^−1^**X**)^−1^  **X***^T^*  **Σ**^−1^ and **Σ** = **K**_GE_*τ*_ge_ + **I**_n_e_n_τ is the variance of **y**. The average information REML (AI-REML) [34] is used to estimate the variance components *τ*_ge_ and *τ*. When the AI-REML algorithm failed to converge, HE regression [33] was used. To circumvent the computational challenges, the PCG was used to indirectly calculate **Σ**^−1^ and perform kinship matrix-related calculation (Text S4).

### Selection of potentially associated marker-by-environment interactions

To obtain potentially associated QTNs (pQTNs) and pQEIs, conditional expectation [31], along with ${\hat{\tau }}_{{\mathrm{ge}}}$ and $\hat{\tau }$ in AI-REML, was used to estimate additive, dominant, additive-by-environment interaction, and dominant-by-environment interaction effects for each locus, while their variances were estimated by GRAMMAR-gamma approximation [32] via parallel computing (Text S5). Wald statistic ${W}_i = {{\boldsymbol \gamma }}_i^{\mathrm{T}}{{{\bf G}}}^{ - 1}{{{\boldsymbol \gamma }}}_i \sim \chi _{df = 2}^2$ for *a_i_* and *d_i_* effects, and ${W}_{{\mathrm{e}}i} = {{\boldsymbol \gamma }}_{{\mathrm{e}}i}^{\mathrm{T}}{{\bf G}}_{\mathrm{e}}^{ - 1}{{{\boldsymbol \gamma }}}_{{\mathrm{e}}i} \sim \chi _{df = 2( {{n}_e - 1} )}^2$ for *ae* and *de* effects for the *i*th locus were then calculated (Text S5), where $${{{\boldsymbol \gamma }}}_i = {( {\begin{array}{*{20}{c}} {{a}_i}&{{d}_i} \end{array}} )}^{\mathrm{T}}$$, $${{{\boldsymbol \gamma }}}_{ie} = {[ {\begin{array}{*{20}{c}} {( {ae} )_i^{\mathrm{T}}}&{( {de} )_i^{\mathrm{T}}} \end{array}} ]}^{\mathrm{T}}$$, and **G** and **G**_e_ were their variance–covariance matrices, respectively. Finally, the loci with *P* values of ≤1.00e−5 (a relaxed threshold that depends on population size) [70,71] were regarded as pQTNs and pQEIs after a de-collinearity process [35].

### Identification of significant QTNs and QEIs using machine learning and likelihood ratio test

All the selected pQTNs/pQEIs were entered into a multi-loci model:


(3) \begin{eqnarray*}
{{\bf y}} = {{\boldsymbol X {\boldsymbol \beta} }} &+& \sum\limits_{i = 1}^{{q}_{\mathrm{QTN}}} {{{{\bf Z}}}_{i{\mathrm{QTN}}}{{{\boldsymbol \gamma }}}_{i{\mathrm{QTN}}}}\\
&&+ \sum\limits_{j = 1}^{{q}_{{\mathrm{QEI}}}} {{{{\bf Z}}}_{j{\mathrm{QEI}}}} {{{\boldsymbol \gamma }}}_{j{\mathrm{QEI}}} + {{\boldsymbol \varepsilon }},
\end{eqnarray*}

where **γ***_i_*_QTN_ is the *a* and *d* vector of the *i*th QTN, and **γ***_i_*_QEI_ is the *ae* and *de* vector of the *j*th QEI. Their effects were estimated by a machine learning algorithm [28] (Text S6). Two key techniques, Woodbury matrix transformation and PCG [29] (Text S6), were implemented to deal with the computational challenge for **Σ**^−1^ in the machine learning process as:


(4) \begin{eqnarray*}
{{{\bf \Sigma }}}^{ - 1} &=& \Big( \sum\limits_{i = 1}^{{q}_{{\mathrm{QTN}}}} {{{{\bf Z}}}_{i{\mathrm{QTN}}}{{\bf Z}}_{i{\mathrm{QTN}}}^{\mathrm{T}}{\tau }_{i{\mathrm{g}}}}\\
&&+ \sum\limits_{j = 1}^{{q}_{{\mathrm{QEI}}}} {{{{\bf Z}}}_{i{\mathrm{QEI}}}{{\bf Z}}_{{\mathrm{jQEI}}}^{\mathrm{T}}{\tau }_{j{\mathrm{ge}}}} + {{\bf I}}\tau \Big)^{ - 1},
\end{eqnarray*}

where *τ_i_*_g_ and *τ_j_*_ge_ are variances for the *i*th QTN and *j*th QEI, respectively. All pQTNs/pQEIs with non-zero effects estimated in (3) are further tested using likelihood ratio statistic. The critical *P* value of 0.05/*m*, based on Bonferroni correction, and the threshold of log odds ratio (LOD) score of ≥3.0 [20] are used to determine significance and suggested QTNs/QEIs, respectively.

### Monte Carlo simulation studies

Based on the work by Li *et al.* [20], the phenotypes of quantitative trait in this study were simulated (Text S2). PLINK v1.9 was used to simulate the genotypic dataset for simulation datasets I, IV and VI. The 1439-rice genotypic dataset [36] was used in simulation datasets II and III. The 1136 Simmental beef cattle genotypic dataset [52] was used in simulation dataset V.

## Supplementary Material

nwag095_Supplemental_File

## Data Availability

The 18K-rice dataset was downloaded from https://figshare.com/s/12978737918eecb74903, and the G2F maize dataset was downloaded from https://doi.org/10.25739/tq5e-ak26. The simulation datasets are available from the corresponding author on request. The Fast3VmrMLM, Fast3VmrMLM-Hap and Fast3VmrMLM-mQTL algorithms were integrated into a multi-environment GWAS module and combined with the existing single-environment GWAS module, resulting in Fast3VmrMLM v2.0, freely available on GitHub (https://github.com/YuanmingZhang65/Fast3VmrMLM). Additional details on the Materials and Methods can be found in the Supplementary data.
