# Supplementary material for: A fast method for breeding by design via G × E interactions detected in large-scale climatic, phenomic and genomic data
Source: Natl Sci Rev. 2026 Feb 11;13(10):nwag095. doi: 10.1093/nsr/nwag095 (PMC13249571; doi:10.1093/nsr/nwag095)
Supplement: nwag095_Supplemental_File [file nwag095_supplemental_file.pdf]

Supplementary Text

S1 The calculation of meteorological factors

The five meteorological factors in the main text were: (1) growing degree days (GGD) (Masle et al. 1989), $GDD = \frac{1}{L}\sum_{i=S}^{S+L}\left[\frac{1}{2}(T_{\max} + T_{\min}) - T_{base}\right]$; (2) photothermal time (PTT) (Masle et al. 1989), $PTT = \frac{1}{L}\left\{\sum_{i=S}^{S+L}\left[\frac{1}{2}(T_{\max} + T_{\min}) - T_{base}\right] \times DL\right\}$; (3) day length (DL); (4) photosynthetically active radiation (PAR) (Yuan et al. 2017) (5) precipitation (PREC) (Zhang et al. 2020), daily amount of rainfall (mm), where $T_{\max}$ and $T_{\min}$ represent the daily maximum and daily minimum temperatures, respectively; T_{base} is a certain threshold base temperature (10 °C) accumulated on a daily basis; S is the day after planting; L represents the length of the window.

Similarly with previous methods [2,40,41], the critical windows of each meteorological factors for FT were determined (Fig. S1, Table S2). In detail, FT was significantly correlated with growing degree days (GDD), photothermal time (PTT) and photosynthetically active radiation (PAR) during the early developmental stages of 1–24 days, 1–21 days and 24–28 days (r = –0.94, –0.93, and –0.85; P = 4.36e–6, 1.34e–5 and 3.99e–4), respectively. During the critical period (floral transition) [42] of FT, FT significantly correlated with day length (DL) at 58–70 days (r=0.66; P=1.90e–2) and precipitation (PREC) at 26–42 days (r=–0.64; P=2.40e–2).

Then, the mean meteorological factor within a given window ranging from the ith day after planting (DAP_i) to the jth DAP_j within 90-day growth period of each environment were calculated and correlated with phenotypes. The windows with the strongest correlation and biological relevance were selected. Then the phenotypes across in 12 environments were regressed on the key meteorological factors in the

windows selected. Finally, the resulting slopes and intercepts were used for GWAS via the QTN detection module of Fast3VmrMLM [35], identifying the QMIs and meteorological factors related GEIs.

REFERENCES

1 Masle J, Doussinault G, Farquhar GD et al. Foliar stage in wheat correlates better to photothermal time than to thermal time. *Plant Cell Environ* 1989; 12:235–247.

2 Yuan HY, Saha S, Vandenberg A et al. Flowering and growth responses of cultivated lentil and wild lens germplasm toward the differences in red to far-red ratio and photosynthetically active radiation. *Front Plant Sci* 2017; 8: 386.

3 Zhang B, Hautier Y, Tan X et al. Species responses to changing precipitation depend on trait plasticity rather than trait means and intraspecific variation. *Funct Ecol* 2020; 34: 2622–33.

S2 Monte Carlo simulation studies

The phenotype vector ($y_{nn_e \times 1}$) of a quantitative trait for n individuals in n_e environments is simulated by: $\mathbf{y}_{nn_e \times 1} = \boldsymbol{\mu} + \sum_{k=1}^{q} (\mathbf{Z}_{ka})_{nn_e \times 1} a_k + \sum_{k=1}^{q} (\mathbf{Z}_{kd})_{nn_e \times 1} d_k + \sum_{k=1}^{q} (\mathbf{Z}_e)_{nn_e \times 1} \# \mathbf{Z}_{ka} (ae)_k + \sum_{k=1}^{q} \mathbf{Z}_e \# \mathbf{Z}_{kd} (de)_k + \mathbf{U} + \boldsymbol{\varepsilon}$, where $\boldsymbol{\mu}$ is the total mean; a_k (additive), d_k (dominant), ae_k (additive-by-environment) and de_k (dominant-by-environment) effects are genetic effects of the kth causal variant, respectively, and are determined by the predetermined heritability (Table S11); q is the number of causal variants; $\mathbf{U}_{nn_e \times 1} \sim (0, \tau \mathbf{K}_{nn_e \times nn_e})$ is the polygenic background; $\boldsymbol{\varepsilon}_{nn_e \times 1} \sim (0, \tau_e \mathbf{I}_{nn_e \times nn_e})$ is the residual error, where τ_e is the residual variance and $\mathbf{I}_{nn_e \times nn_e}$ is the Identity matrix. The heritability of the k-th causal variant is calculated by $h^2 = (\varphi_{ka} + \varphi_{kd} + \varphi_{kae} + \varphi_{kde}) / (\varphi_a + \varphi_d + \varphi_{ae} + \varphi_{de} + \tau + \tau_e)$, where φ_{ka} and φ_{kd} are the additive and dominant variances of the *kth* causal variant, respectively, and φ_{kae} and φ_{kde} are additive-by- and dominant-by- environment variances of the *kth* causal variant, respectively; φ_a, φ_d, φ_{ae} and φ_{de} are the total variances for corresponding effects of all causal variants.

In simulation dataset I, 1,000 individuals with 100,000 SNPs each in two environments were simulated using the PLINK v1.9 [54] and 10 causal variants with heritability ranging from 5% to 10% were set. Among the 10 variants, 1 had dominant effect only and 2 had dominant-by-environment effect only (Table S11). Four polygenic backgrounds were simulated: none (I-1), additive-dominant (I-2), epistatic (I-3) and additive-dominant-epistatic (I-4). The number of replicates was 100.

In simulation dataset II, 600 individuals with 100,000 SNPs each, are randomly selected from 1,439 rice dataset in [36] and their phenotypes in three environments were simulated with four types of polygenic backgrounds: none (II-1), additive-dominant (II-2), epistatic (II-3) and additive-dominant-epistatic (II-4). Causal variants with heritability of 5% to 10% were simulated, and their parameters are listed in Table S15. The number of replicates was 100.

In simulation dataset III, with the genotype of the simulation II (600 individuals with

100,000 SNPs). There were 10% polygenic background but no causal variants were set. Thus, the phenotypes were determined only by the polygenic background and the residual term. The number of replicates was 1,000.

In simulation dataset IV, we aimed to investigate the consumption of computational resources for Fast3VmrMLM in various sample size (n) and environment numbers (n_e). Three series simulations were conducted in simulation IV. For large n, PLINK v1.9 was used to simulate 1,000, 5,000, 10,000, 15,000 and 20,000 sample size each with one million SNPs in two environments in simulation IV-1. For large n_e, PLINK v1.9 was used to simulate the n_e ranging from 8 to 40 in simulation IV-2 with 1,000 individuals each with 1,000,000 SNPs. For both large n and large n_e, 20,000 individuals with one million SNPs in 20 environments and 5,000 individuals with one million SNPs in 40 environments were simulated in simulation IV-3.

In simulation dataset V, 1,136 Simmental beef cattle with all 669,742 markers in [56] were used. The haplotypes with the heritability ranging from 8% to 10% were used to simulate the functional elements in the real genome. The 7th and 8th variants had rare SNPs (MAF = 3%), as well as 9th and 10th variants had rare SNPs (MAF = 1%). The number of replicates was 100.

In simulation dataset VI, the genotype of simulation dataset I was used to construct the structure variants (SVs) by combining adjacent three SNPs. As a results, 1,000 individuals each with 33,341 SVs were constructed. There were 4 causal SVs and 6 causal SV-by-environment interactions ranging from 5% to 10% heritability. The number of replicates was 100.

S3 The full mixed linear model for QTNs and QEIs detection

The full mixed linear model (MLM) detecting QTNs and QEIs for the *i*th marker in multi-environment datasets is indicated by

$$\begin{aligned}\mathbf{y} = \mathbf{X\beta} + \left(\mathbf{Z}_e\right)_{n_e n\times(n_e-1)} \mathbf{e}_{(n_e-1)\times 1} + \left(\mathbf{Z}_{ia}\right)_{n_e n\times 1} a_i + \left(\mathbf{Z}_{id}\right)_{n_e n\times 1} d_i + \left(\mathbf{Z}_e \# \mathbf{Z}_{ia}\right)_{n_e n\times(n_e-1)} (ae)_i \\ + \left(\mathbf{Z}_e \# \mathbf{Z}_{id}\right)_{n_e n\times(n_e-1)} (de)_i + \mathbf{U}_a + \mathbf{U}_d + \mathbf{U}_{ae} + \mathbf{U}_{de} + \boldsymbol{\varepsilon}\end{aligned} \quad (1)$$

where $\mathbf{y}_{n_e n\times 1}$ is the phenotype vector with $n_e \times n$ individuals; $\mathbf{X}$ is the $n_e n\times q$ incident matrix of fixed effects $\boldsymbol{\beta}_{q\times 1}$; $\mathbf{Z}_e$ is the $n_e n\times(n_e-1)$ incident matrix of environment effects $\mathbf{e}_{(n_e-1)\times 1}$; a_i (d_i) are the additive (dominant) effect of the *i*th marker; $(ae)_i$ and $(de)_i$ are additive-by-environment and dominant-by-environment interaction effects of the *i*th marker, respectively; $\mathbf{Z}$ is the design matrix of corresponding effects; $\mathbf{U}_a \sim \text{MVN}(0,\tau_a\mathbf{K}_a)$, $\mathbf{U}_d \sim \text{MVN}(0,\tau_d\mathbf{K}_d)$, $\mathbf{U}_{de} \sim \text{MVN}(0,\tau_{ae}\mathbf{K}_{ae})$ and $\mathbf{U}_{de} \sim \text{MVN}(0,\tau_{de}\mathbf{K}_{de})$ are polygenic background vectors, respectively, where τ_a, τ_d, τ_{ae} and τ_{de} are additive, dominant, additive-by-environment and dominant-by-environment polygenic variances, respectively, where $\mathbf{K}_a=\frac{1}{m}\left(\mathbf{Z}_a\mathbf{Z}_a^{\mathrm{T}}\right)$, $\mathbf{K}_d=\frac{1}{m}\left(\mathbf{Z}_d\mathbf{Z}_d^{\mathrm{T}}\right)$, $\mathbf{K}_{ae}=\frac{1}{m}\left[\left(\mathbf{Z}_a \# \mathbf{Z}_e\right)\left(\mathbf{Z}_a \# \mathbf{Z}_e\right)^{\mathrm{T}}\right]$ and $\mathbf{K}_{de}=\frac{1}{m}\left[\left(\mathbf{Z}_d \# \mathbf{Z}_e\right)\left(\mathbf{Z}_d \# \mathbf{Z}_e\right)^{\mathrm{T}}\right]$; $\boldsymbol{\varepsilon}\sim\text{MVN}(0,\mathbf{I}_{n_e n}\tau)$ is residual error, where τ is residual variance and $\mathbf{I}_{n_e n}$ is the $n_e n$ dimensional identity matrix.

S4 AI-REML and Preconditioned Conjugate Gradient (PCG)

The REML function of model (2) given τ_{ge} and τ was:

$$l_R(\tau_{ge},\tau) \propto -\frac{1}{2}\left[(n-q)\log(\tau_{ge}) + \log(\boldsymbol{\Sigma}) + \log\left|\mathbf{X}^{\mathrm{T}}\boldsymbol{\Sigma}^{-1}\mathbf{X}\right| + \frac{1}{\tau}\mathbf{y}^{\mathrm{T}}\mathbf{P}\mathbf{y}\right] \quad (2)$$

where $\mathbf{P} = \boldsymbol{\Sigma}^{-1} - \boldsymbol{\Sigma}^{-1}\mathbf{X}\left(\mathbf{X}^{\mathrm{T}}\boldsymbol{\Sigma}^{-1}\mathbf{X}\right)^{-1}\mathbf{X}^{\mathrm{T}}\boldsymbol{\Sigma}^{-1}$ and $\boldsymbol{\Sigma} = \mathbf{K}_{GE}\tau_{ge} + \mathbf{I}_{n_e n}\tau$ is the variance of $\mathbf{y}$. Based on the likelihood function (3) we used AI-REML [34] to iteratively estimate the variance components τ_{GE} and τ via $\hat{\tau}_{\mathrm{GE}}^{(t+1)} = \hat{\tau}_{\mathrm{GE}}^{(t)} + \left(\mathrm{AI}_{\mathrm{GE}}^{(t)}\right)^{-1}\left[\partial l_{\mathrm{R}}\left(\hat{\tau}_{\mathrm{GE}}^{(t)}\right)/\partial\tau_{\mathrm{GE}}\right]$ and $\hat{\tau}^{(t+1)} = \hat{\tau}^{(t)} + \left(\mathrm{AI}^{(t)}\right)^{-1}\left[\partial l_{\mathrm{R}}\left(\hat{\tau}^{(t)}\right)/\partial\tau\right]$ at the t-th iteration, where $\mathrm{AI}_{\mathrm{GE}}^{(t)} = 0.5\mathbf{y}^{\mathrm{T}}\mathbf{P}\mathbf{K}_{\mathrm{GE}}\mathbf{P}\mathbf{K}_{\mathrm{GE}}\mathbf{P}\mathbf{y}$, $\mathrm{AI}^{(t)} = 0.5\mathbf{y}^{\mathrm{T}}\mathbf{P}\mathbf{I}\mathbf{P}\mathbf{I}\mathbf{P}\mathbf{y}$, $\partial l_{\mathrm{R}}\left(\hat{\tau}_{\mathrm{GE}}^{(t)}\right)/\partial\tau_{\mathrm{GE}} = 0.5\left[\mathbf{y}^{\mathrm{T}}\mathbf{P}\mathbf{K}_{\mathrm{GE}}\mathbf{P}\mathbf{y} - \mathrm{tr}\left(\mathbf{P}\mathbf{K}_{\mathrm{GE}}\right)\right]$ and $\partial l_{\mathrm{R}}\left(\hat{\tau}^{(t)}\right)/\partial\tau = 0.5\left[\mathbf{y}^{\mathrm{T}}\mathbf{P}\mathbf{I}\mathbf{P}\mathbf{y} - \mathrm{tr}\left(\mathbf{P}\mathbf{I}\right)\right]$. Once $\hat{\tau}_{\mathrm{GE}}^{(t+1)}$ and $\hat{\tau}^{(t+1)}$ for the (t+1)th iteration is obtained, $\hat{\boldsymbol{\beta}}^{(t+1)}$ can be estimated from $\hat{\boldsymbol{\beta}} = \left(\mathbf{X}^{\mathrm{T}}\boldsymbol{\Sigma}^{-1}\mathbf{X}\right)^{-1}\mathbf{X}^{\mathrm{T}}\boldsymbol{\Sigma}^{-1}\mathbf{y}$. The iteration of AI-REML ends until $\hat{\boldsymbol{\beta}}$, τ_{GE} and τ converge. To circumvent the computational challenges posed by directly inverting the variance-covariance matrix $\boldsymbol{\Sigma}$ in each iteration of AI-REML, particularly in large-scale datasets, the Preconditioned Conjugate Gradient (PCG) method [29] was utilized as the follow.

In each iteration of AI-REML, the expression form $\boldsymbol{\Sigma}^{-1}\boldsymbol{\phi}$ should be updated, which is a challenging for large-scale dataset. To circumvent the computational challenge, PCG [29, 55] is used to calculate $(\boldsymbol{\Sigma})^{-1}$ indirectly. In the iteration formula of $\boldsymbol{\Sigma}\boldsymbol{\phi}_{\mathrm{y}} = \mathbf{y}$, we update $\boldsymbol{\phi}_{\mathrm{y}}$ so that the inverse matrix is calculated indirectly via $\hat{\boldsymbol{\Sigma}}^{-1}\mathbf{y} = \boldsymbol{\phi}_{\mathrm{y}}$. Here the computational complexity is reduced from $O(n^3)$ to $O(Pn^2)$, where P is the number of iterations in PCG and n is the total individual number among all environments, which is less than 10 on average in [55]. In addition, when the sample size is larger than forty thousand, the strategy of calculating kinship matrix in each iteration of PCG which was described in [35]. This strategy eliminates the need to store the entire kinship matrix, especially for large cohorts.

S5 The conditional expectation and GRAMMAR-gamma approximation

The conditional expectation was used to estimate all QTN and QEI effects by $a_i = \frac{\tau_{GE}}{2m+(n_e-1)m}\mathbf{Z}_{ia}{}^{\mathrm{T}}\mathbf{Py}$, $d_i = \frac{\tau_{GE}}{2m+(n_e-1)m}\mathbf{Z}_{id}{}^{\mathrm{T}}\mathbf{Py}$, $(ae)_i = \frac{\tau_{GE}}{2m+(n_e-1)m}(\mathbf{Z}_{ia}\#\mathbf{Z}_e)^{\mathrm{T}}\mathbf{Py}$, $(de)_i = \frac{\tau_{GE}}{2m+(n_e-1)m}(\mathbf{Z}_{id}\#\mathbf{Z}_e)^{\mathrm{T}}\mathbf{Py}$, and corresponding variance by $diag(\mathrm{var}(a_i)) = \left[\frac{\tau_{GE}}{2m+(n_e-1)m}\right]^2 \sum_l^n \left[\mathbf{Z}_{ia}{}^{\mathrm{T}}\mathbf{P}\mathbf{Z}_{ia}^{\mathrm{T}}\right]_{.l}$, $diag(\mathrm{var}(d_i)) = \left[\frac{\tau_{GE}}{2m+(n_e-1)m}\right]^2 \sum_l^n \left[\mathbf{Z}_{id}{}^{\mathrm{T}}\mathbf{P}\mathbf{Z}_{id}^{\mathrm{T}}\right]_{.l}$, $diag(\mathrm{var}(ae_i)) = \left[\frac{\tau_{GE}}{2m+(n_e-1)m}\right]^2 \sum_l^n \left[(\mathbf{Z}_{ia}\#\mathbf{Z}_e)^{\mathrm{T}}\mathbf{P}(\mathbf{Z}_{ia}\#\mathbf{Z}_e)^{\mathrm{T}}\right]_{.l}$ and $diag(\mathrm{var}(de_i)) = \left[\frac{\tau_{GE}}{2m+(n_e-1)m}\right]^2 \sum_l^n \left[(\mathbf{Z}_{id}\#\mathbf{Z}_e)^{\mathrm{T}}\mathbf{P}(\mathbf{Z}_{id}\#\mathbf{Z}_e)^{\mathrm{T}}\right]_{.l}$, where # is the Hadamard product and $\sum_i^n (\mathbf{A}_{n\times n})_{\bullet i}$ represent the row sum of $\mathbf{A}$.

The GRAMMAR-gamma approximation method [32] is used to estimate the variance and covariance of all markers: 1) 30 variants with minor allele counts greater than 20 [55] are randomly selected on the whole genome to estimate the gamma ratio ($\hat{\gamma}_a$, $\hat{\gamma}_d$, $\hat{\gamma}_{ae}$ and $\hat{\gamma}_{de}$) via $\hat{\gamma}_a = \frac{1}{J}\sum_{j=1}^J \frac{\tilde{\mathbf{Z}}_{ja}^{\mathrm{T}}\hat{\mathbf{P}}\tilde{\mathbf{Z}}_{ja}}{\tilde{\mathbf{Z}}_{ja}^{\mathrm{T}}\tilde{\mathbf{Z}}_{ja}}$, $\hat{\gamma}_d = \frac{1}{J}\sum_{j=1}^J \frac{\tilde{\mathbf{Z}}_{jd}^{\mathrm{T}}\hat{\mathbf{P}}\tilde{\mathbf{Z}}_{jd}}{\tilde{\mathbf{Z}}_{jd}^{\mathrm{T}}\tilde{\mathbf{Z}}_{jd}}$, $\hat{\gamma}_{ae} = \frac{1}{J}\sum_{j=1}^J \frac{\tilde{\mathbf{Z}}_{jae}^{\mathrm{T}}\hat{\mathbf{P}}\tilde{\mathbf{Z}}_{jae}}{\tilde{\mathbf{Z}}_{jae}^{\mathrm{T}}\tilde{\mathbf{Z}}_{jae}}$ and $\hat{\gamma}_{de} = \frac{1}{J}\sum_{j=1}^J \frac{\tilde{\mathbf{Z}}_{jde}^{\mathrm{T}}\hat{\mathbf{P}}\tilde{\mathbf{Z}}_{jde}}{\tilde{\mathbf{Z}}_{jde}^{\mathrm{T}}\tilde{\mathbf{Z}}_{jde}}$, where $\tilde{\mathbf{Z}} = \mathbf{Z} - \mathbf{X}(\mathbf{X}^{\mathrm{T}}\mathbf{X})^{-1}\mathbf{X}^{\mathrm{T}}\mathbf{Z}$ and $\hat{\mathbf{P}} = \mathbf{\Sigma}^{-1}\mathbf{X}(\mathbf{X}^{\mathrm{T}}\mathbf{\Sigma}^{-1}\mathbf{X})^{-1}\mathbf{X}^{\mathrm{T}}\mathbf{\Sigma}^{-1}$; 2) the variances and covariances for the main and interactive effects are approximated using $diag(Var(\mathbf{a})) \approx \hat{\gamma}_a \left[\frac{\tau_{GE}}{2m+(n_e-1)m}\right]^2 \sum_i^n (\tilde{\mathbf{Z}}_a^{\mathrm{T}}\#\tilde{\mathbf{Z}}_a^{\mathrm{T}})_{\bullet i}$, $diag(Var(\mathbf{d})) \approx \hat{\gamma}_a \left[\frac{\tau_{GE}}{2m+(n_e-1)m}\right]^2 \sum_i^n (\tilde{\mathbf{Z}}_d^{\mathrm{T}}\#\tilde{\mathbf{Z}}_d^{\mathrm{T}})_{\bullet i}$, $diag(Var(\mathbf{ae})) \approx$

$\hat{\gamma}_{ae}\left[\frac{\tau_{\text{GE}}}{2m+(n_e-1)m}\right]^2 \sum_i^n\left[\left(\tilde{\mathbf{Z}}_a^{\text{T}}\#\mathbf{Z}_e^{\text{T}}\right)\#\left(\tilde{\mathbf{Z}}_a^{\text{T}}\#\mathbf{Z}_e^{\text{T}}\right)\right]_{\bullet i}$, and $diag\left(Var\left(\mathbf{de}\right)\right) \approx$ $\hat{\gamma}_{ae}\left[\frac{\tau_{\text{GE}}}{2m+(n_e-1)m}\right]^2 \sum_i^n\left[\left(\tilde{\mathbf{Z}}_d^{\text{T}}\#\mathbf{Z}_e^{\text{T}}\right)\#\left(\tilde{\mathbf{Z}}_d^{\text{T}}\#\mathbf{Z}_e^{\text{T}}\right)\right]_{\bullet i}$. The covariances of above effects have similarly approximate. Hence, the computational complexity is reduced from $O\left(mN^2\right)$ to $O\left(mN\right)$. where m is the marker number and N is the total individual number among all environments.

Based on above effects and variance-covariance matrices, the vectorized Wald test was used to calculate the Wald statistic W_i of the ith marker using $W_i = \boldsymbol{\gamma}_i^{\text{T}}\mathbf{G}^{-1}\boldsymbol{\gamma}_i \sim \chi^2_{df=2}$, where $\boldsymbol{\gamma}_i = \left(a_i \quad d_i\right)^{\text{T}}$ and $\mathbf{G}$ was the variance-covariance matrix of $\boldsymbol{\gamma}_i$ [35]. The Wald statistic W_{ei} of the ith marker-by-environment effect using $W_{ei} = \boldsymbol{\gamma}_{ei}^{\text{T}}\mathbf{G}_e^{-1}\boldsymbol{\gamma}_{ei} \sim \chi^2_{df=2(n_e-1)}$, where $\boldsymbol{\gamma}_{ie} = \left[(ae)_i^{\text{T}} \quad (de)_i^{\text{T}}\right]^{\text{T}}$ and $\mathbf{G}_e$ was the variance-covariance matrix of $\boldsymbol{\gamma}_{ie}$.

S6 The expectation-maximization empirical Bayes (EM empirical Bayes) method

All the q_{QTN} and q_{QEI} selected pQTNs and pQEIs were entered into model (4):

$$\mathbf{y} = \mathbf{X}\boldsymbol{\beta} + \sum_{i=1}^{q_{\text{QTN}}} \mathbf{Z}_{i\text{QTN}}\boldsymbol{\gamma}_{i\text{QTN}} + \sum_{j=1}^{q_{\text{QEI}}} \mathbf{Z}_{j\text{QEI}}\boldsymbol{\gamma}_{j\text{QEI}} + \boldsymbol{\varepsilon} \tag{4}$$

where $\boldsymbol{\gamma}_{i\text{QTN}}$ is the additive and dominant effect vector of the ith QTNs, $\boldsymbol{\gamma}_{\text{jQEI}}$ is the *ae* and *de* effect vector of the jth QEIs. EM empirical Bayes was used to estimate all the main and interactive effects. The major limitation of applying EM empirical Bayes on large-scale dataset is the need to compute the inverse of a large matrix $\mathbf{\Sigma}$ via equation (1):

$$\mathbf{\Sigma}^{-1} = \left(\sum_{i=1}^{q_{\text{QTN}}} \mathbf{Z}_{i\text{QTN}}\mathbf{Z}_{i\text{QTN}}^{\text{T}}\tau_{ig} + \sum_{j=1}^{q_{\text{QEI}}} \mathbf{Z}_{i\text{QEI}}\mathbf{Z}_{\text{jQEI}}^{\text{T}}\tau_{jge} + \mathbf{I}\tau\right)^{-1} \tag{1}$$

where τ_{ig} is the ith QTN variance and τ_{jge} is the jth QEI variance, which significantly increases the computational complexity and memory requirements. The main computational complexities in equation (1) are the $O\left(qn^2\right)$ of marker-related term and the $O\left(n^3\right)$ of the inverse of $\mathbf{\Sigma}$. To address these issues, we implemented two key techniques: Woodbury matrix transformation and PCG [29].

When the number of potentially associated QTNs and QEIs ($q_{\text{QTN}} + q_{\text{QEI}}$) is less than the sample size ($n_e n$), we have the Woodbury matrix transformation for $\mathbf{\Sigma}^{-1}$:

$$\begin{aligned}\mathbf{\Sigma}^{-1} &= \left(\sum_{l=1}^{l=L} \mathbf{Z}_l\mathbf{Z}_l^{\text{T}}\tau_l + \mathbf{I}\tau\right)^{-1} \\ &= \left(\mathbf{ZCZ}^T + \mathbf{A}\right)^{-1} \\ &= \mathbf{A}^{-1} - \mathbf{A}^{-1}\mathbf{Z}\left(\mathbf{C}^{-1} + \mathbf{Z}^T\mathbf{A}^{-1}\mathbf{Z}\right)^{-1}\mathbf{Z}^T\mathbf{A}^{-1}\end{aligned} \tag{5}$$

where $\mathbf{Z}_l$ is the design matrix of variance τ_l in (1) for the lth QTN or QEI, $\mathbf{C}$ is a diagonal matrix with the diagonal element $\mathbf{C}_{ll} = \tau_l$ and $\mathbf{A} = \mathbf{I}_{n_e n}\tau$. The Woodbury matrix transformation is used at the third equal sign. Thus, the computational complexity of the tth iteration is reduced from $O\left(qm^2 + m^3\right)$ to $O\left(mq^2 + q^3\right)$, where $q = q_{\text{QTN}} + q_{\text{QEI}}$ and $m = n_e n$. It is clear that when $q < m$, the

computational complexity is reduced.

When both of the q and m are greater than four thousand, the PCG method is used to calculate the $\boldsymbol{\Sigma}^{-1}$ in the same way as in Text S1. The above two techniques realized the fast and cheap EM empirical Bayes framework.
